# Use of “Entertainment” Chimpanzees in Commercials Distorts Public Perception Regarding Their Conservation Status

**DOI:** 10.1371/journal.pone.0026048

**Published:** 2011-10-12

**Authors:** Kara K. Schroepfer, Alexandra G. Rosati, Tanya Chartrand, Brian Hare

**Affiliations:** 1 Department of Evolutionary Anthropology, Duke University, Durham, North Carolina, United States of America; 2 Center for Cognitive Neuroscience, Duke University, Durham, North Carolina, United States of America; 3 Fuqua School of Business, Duke University, Durham, North Carolina, United States of America; 4 Department of Psychology and Neuroscience, Duke University, Durham, North Carolina, United States of America; Université Pierre et Marie Curie, France

## Abstract

Chimpanzees (*Pan troglodytes*) are often used in movies, commercials and print advertisements with the intention of eliciting a humorous response from audiences. The portrayal of chimpanzees in unnatural, human-like situations may have a negative effect on the public's understanding of their endangered status in the wild while making them appear as suitable pets. Alternatively, media content that elicits a positive emotional response toward chimpanzees may increase the public's commitment to chimpanzee conservation. To test these competing hypotheses, participants (n = 165) watched a series of commercials in an experiment framed as a marketing study. Imbedded within the same series of commercials was one of three chimpanzee videos. Participants either watched 1) a chimpanzee conservation commercial, 2) commercials containing “entertainment” chimpanzees or 3) control footage of the natural behavior of wild chimpanzees. Results from a post-viewing questionnaire reveal that participants who watched the conservation message understood that chimpanzees were endangered and unsuitable as pets at higher levels than those viewing the control footage. Meanwhile participants watching commercials with entertainment chimpanzees showed a decrease in understanding relative to those watching the control footage. In addition, when participants were given the opportunity to donate part of their earnings from the experiment to a conservation charity, donations were least frequent in the group watching commercials with entertainment chimpanzees. Control questions show that participants did not detect the purpose of the study. These results firmly support the hypothesis that use of entertainment chimpanzees in the popular media negatively distorts the public's perception and hinders chimpanzee conservation efforts.

## Introduction

Chimpanzees in the wild face an uncertain future due to habitat destruction, disease, and hunting fueled by the illegal pet trade [1]. As a result, all African countries in which chimpanzees live forbid their capture and trade as food or as pets (CITES www.cites.org; and African Convention on the Conservation of Nature and Natural Resources). However, chimpanzees are the only primate species with two listings on the Endangered Species List in the United States. Wild-born chimpanzees are listed as endangered while individuals captive-born in the U.S. are only listed as threatened [2]. This split listing was designated to give the U.S. Fish and Wildlife Service the authority to prevent the importation of wild chimpanzees from Africa but simultaneously allow for the use and trade of U.S. born captive chimpanzees. As a result, while it is illegal for an African citizen to trade or own a chimpanzee anywhere in Africa, chimpanzees can be privately purchased by individuals or corporations in the U.S. for the purpose of keeping them as pets, using them as entertainment, or for the purpose of biomedical research.

For decades animal trainers have bought, bred and trained chimpanzees as entertainers for use in print advertisements, greeting cards, television advertisements, television shows, or movies [3]. Typically, infant and juvenile chimpanzees are used while still physically manageable and maximally attractive to viewers [4], [5]. As a consequence, a constant supply of infants is needed even though chimpanzees too old to entertain must be provided life-time care for up to sixty years. A number of animal welfare groups have actively campaigned to end the use of privately owned chimpanzees for entertainment due to documented welfare concerns [6], [7]. However, no one has ever experimentally assessed how video stimuli of entertainment chimpanzees affects the perceptions of the wider public regarding their conservation status in the wild and suitability as pets.

Two hypotheses predict different effects concerning how viewing of entertainment chimpanzees influence public perceptions of chimpanzees more generally, First, the *distortion hypothesis* suggests that the regular appearance of trained chimpanzees in commercials and other forms of media lead the public to believe chimpanzees are not endangered and potentially make appropriate pets. The public's perceptions of non-endemic species are largely informed by what they see in the media, museums, zoos and aquariums [8]. Visits to local zoos are relatively infrequent with few zoos exhibiting chimpanzees while popular media is saturated with entertainment chimpanzees; 35 commercials, 15 TV episodes and 7 movies featured chimpanzees in human-like situations in the last five years [3]. This includes commercials during events with huge national audiences such as the super bowl. Given that the U.S. public is most often exposed to chimpanzees as entertainers, this could lead many to believe that this species is not threatened – otherwise it would not be used this way on television. In support of this hypothesis, a survey conducted by Ross et al. [9] at the Lincoln Park Zoo in Chicago revealed that 33% of respondents who correctly indicated that orangutans and gorillas were endangered failed to indicate that chimpanzees were endangered. When asked to specify why, the most common response was that chimpanzees were frequently seen on TV making it unlikely they were endangered. In an experimental follow-up, Ross et al [10] found that when observing photographs of chimpanzees in different situations viewers were less likely to indicate chimpanzees were endangered and more suitable as pets when both a chimpanzee and a human appeared in the photograph or if a chimpanzee appeared in an anthropomorphic setting such as an office. This confusion may impact the willingness of the public to support chimpanzee conservation efforts as uncertainty regarding a species status in the wild has been documented to reduce public financial contributions or willingness-to-pay (WTP) [11].

Chimpanzees in commercials and movies, dressed and behaving like humans, may also distort the public's perception of chimpanzees as pets, perpetuating the false belief that they are suitable household companions. In the United States, there are an estimated 15,000 primates kept as pets [7], including over 300 chimpanzees in households and private zoos [3]. Despite media coverage of horrific attacks by privately owned chimpanzees, a number of individuals still actively breed and sell chimpanzees for private ownership in the United States. The presentation of trained entertainment chimpanzees interacting seamlessly with humans in edited footage can only encourage the belief of potential customers that chimpanzees can provide an exotic, almost human, form of companionship. Worse yet, the presence of entertainment chimpanzees on television may increasingly have an effect on conservation efforts in chimpanzee range countries. The potential profits from the illegal sale of infant apes is a driving force behind the poaching of wild ape populations [12]–[14]. The majority of these infants are bought by expatriates who have likely been primed by entertainment chimpanzees that appear as suitable pets (although a significant number are also illegally exported). Meanwhile, through the expansion of satellite TV and the importation of foreign films, western media is increasingly available in African cities and new export markets in the Middle East and Asia where regulations on wildlife trafficking can be lax. The depiction of westerners on television or in print media interacting with chimpanzees as if they are pets can only further encourage the belief of poachers and animal traffickers that expatriates and foreigners have a strong desire for infant chimpanzees [15]. Of course, one viral video could accidently turn this perceived international demand for pet apes into a reality. Even a small increase in demand – even as a short fad – could spell doom for crashing wild populations.

Alternatively, as many media outlets privately argue when internally justifying the use of privately owned chimpanzees, the presence of chimpanzees in the media, in any form, may enhance conservation efforts by reminding the public of their presence and likability [16]. The *familiarity hypothesis* suggests that any presentation of chimpanzees helps conservation efforts – even when presented in unnatural or human-like situations. Indeed research does suggest that familiarity is a factor that can influence the public's support of conservation organizations, along with personal reputation, biophilia, personal income, knowledge and interests [17]–[22]. Moreover, people's donation preferences are also positively correlated with the degree to which a species is similar to humans [23]–[25] with chimpanzees having a high baseline likability index because of their close relationship to humans [24]. Therefore, media representations of chimpanzees behaving like humans while wearing clothes could enhance the public's tendency to support chimpanzee conservation.

In order to test between the predictions of the distortion and familiarity hypothesis we exposed 165 participants to a series of commercials that included either 1) a conservation message about chimpanzees 2) entertainment chimpanzees in commercials or 3) a control video with wild chimpanzees behaving naturally. Participants were then given a questionnaire about all the commercials which included a few embedded target questions about chimpanzees. They were also given a chance to donate a portion of their participation payment to a conservation organization. The distortion hypothesis predicts that relative to a baseline condition those watching a conservation message will have more accurate knowledge and donate more to conservation while entertainment chimpanzee commercials will have a negative impact. The familiarity hypothesis predicts that relative to a baseline both the conservation and the entertainment chimpanzee commercials will have a positive effect.

## Methods

### Ethics Statement

The experimental protocols were approved by Duke University Health System's Institutional Review Board, protocol ID pro00015168 and all participants signed a written consent form before the start of the experiment.

### Experiment 1

#### Data Collection

Duke University students and affiliated members were recruited through poster advertisements to participate in a marketing study. Fifteen sessions of 2 – 7 participants were conducted between October and December 2009. Participants sat at a private computer, signed a consent form and proceeded through a 7.5 minute video clip and an 87 question survey. Experimenters monitored participants' progress and as they finished participants were asked if they would like to donate part of their experimental earnings to a particular charity and/or purchase products that they viewed in the commercials. Participants (n = 36) were randomly assigned to 1 of 3 conditions: Public Service Announcement (PSA), Baseline or Hollywood.

#### Videos

Videos were acquired through YouTube in September of 2009 and compiled into one clip using iMovie software. All three conditions contained the same decoy commercials: Coca-Cola (2009), Crest toothpaste (2008), Aquafresh toothpaste (2008) and Save the Children (2008). One of the following test stimuli was embedded within the decoy commercials in each of the three test conditions: (1) Public Service Announcement (PSA) – Jane Goodall Institute (2009; Jane Goodall and others describe the threats facing chimpanzees), (2) Baseline – Mahale Mountains (2009: video of natural behavior of wild chimpanzees in Mahale National Park), (3) Hollywood – Career Builder (2006; HR employee interviews a series of chimps), E*trade (2000; chimpanzee dances to music) & Spirit Bank (2008; chimpanzee works in office). Commercial length was controlled; product commercials totaled 1 minute in length and charity commercials and test stimuli were 2.5 minutes in length. In each of the three conditions the order in which the different commercials were presented was counterbalanced. See http://www.duke.edu/~ks163/Chimpanzee_conservation_media/ for stimuli.

#### Survey

The survey assessed participants' attitudes and knowledge towards chimpanzees in three major areas: their suitability as pets, presence in the media and survival in the wild (see Table 1). To disguise the aim of the study, forty six questions regarding the decoy stimuli were included in addition to ten target questions on chimpanzees or great apes. Thirty-one questions were control questions and assessed subjects' demographics, access to information, donation history and environmental habits. The Personal Altruism Scale [26] was included to control for potential altruistic biases. Question format was variable and included scale, yes/no, multiple choice and check-box questions. Surveys were identical across conditions with the exception of three questions where potential response choices were altered given the test video the subject watched.

**Table 1:** Target Question Results from Experiment 1 & 2.

		Experiment 1		Experiment 2	
Question	Format	Means	Result	Means	Result
**PET COMPOSITE**	**Composite**	**2.79, 2.85, 2.31**	**P>B, B>H**	**3.03, 2.86, 2.53**	**P>H**
Would you consider having a chimpanzee as a pet?	Y/N	0.00, 0.00, 0.08	NS	-	-
Is a chimpanzee too large to be a pet?	Y/N	0.92, 0.92, 0.58	NS	-	-
Do you agree: Exotic animals make good pets?	Scale 1- 5	1.50, 1.25, 1.75	NS	-	-
Where in the US is it illegal to have a chimpanzee as a pet?	MC	0.33, 0.67, 0.75	NS	-	-
How big is an adult chimpanzee?	MC	-	-	0.36, 0.28, 0.19	NS
People should have the right to buy and keep a chimpanzee if they want to?	Y/N	-	-	0.09, 0.12, 0.35	***
If your friend wanted a chimpanzee as a pet would you be supportive?	Y/N	-	-	0.07, 0.12, 0.14	NS
Do you agree: Primates such as chimpanzees make good pets?	Scale 1 – 5	-	-	1.68, 1.74, 1.65	NS
**ENTERTAINMENT COMPOSITE**	**Composite**	**8.00, 6.17, 7.17**	**P>B**	**7.77, 7.14, 7.12**	**P>B, P>H**
Do you agree: Chimpanzees are unintelligent?	Scale 1 – 5	1.42, 2.17, 1.67	NS	1.86, 2.19, 2.02	NS
Do you agree: It harms animals to train and use them in the media?	Scale 1 – 5	3.42, 2.33, 2.83	P>B	3.64, 3.33, 3.14	NS
**ENDANGERED STATUS COMPOSITE**	**Composite**	**1.83, 1.08, 0.83**	**P>B, P>H**	**1.82, 1.07, 0.98**	**P>B, P>H**
How many chimpanzees remain in the wild?	MC	0.92, 0.58, 0.50	NS	0.84, 0.53, 0.58	***
Is the chimpanzee endangered?	Y/N	0.92, 0.50, 0.33	**	0.98, 0.53, 0.40	***
Is the gorilla endangered?†	Y/N	0.58, 0.25, 0.42	NS	0.50, 0.44, 0.40	NS
Is the orangutan endangered?†	Y/N	0.50, 0.33, 0.25	NS	0.45, 0.37, 0.37	NS
**DONATION**					
If given $50 to divide between the Red Cross and the World Wildlife Fund how much would you give to each?	DC	-	-	17.4, 16.4, 13.5	NS
Proportion Donating (Exp 1: BCTF, Exp 2: AWF)	Y/N	0.25, 0.00, 0.00	*	0.23, 0.14, 0.07	NS
Amount Donated (Exp 1:BCTF, Exp 2: AWF)	NR	0.67, 0.00, 0.00	NS	0.52, 0.51, 0.19	NS

Summary of answers to target questions. Question format, means for each condition and statistical results are indicated for Experiment 1 and Experiment 2.

Format: Composite- combination of all questions listed below bold heading (see text for calculations), Y/N – Yes or No question, Scale 1 – 5 – Rate answer on a fixed scale, MC – multiple choice, DC – dichotomous choice, NR – numerical response. – indicates question was not asked in experiment. Result: P – PSA condition, B – Baseline condition, H – Hollywood condition , NS – not significant

*denotes p<0.05 in Chi^2^.

**denotes p<0.02 in Chi^2^

***denotes p<0.01 in Chi^2^

†Question not included in the composite score.

#### Donations

After the survey, participants received a handout where they indicated if they wished to purchase a 12 oz can of Coca-Cola, a 1 oz tube of Crest or Aquafresh toothpaste and/or donate to American Red Cross and/or a conservation organization (Bushmeat Crisis Task Force). These charities were selected to mirror the organizations seen in the video and their respective aims were explained on the handout. Participants were also prompted verbally by the experimenter to confirm their choice to donate or not to donate a portion of their experimental payment to these organizations. Payment for participation was via check and donations and purchases were deducted from the participation payment of $10.

#### Analysis

For the purpose of analysis the ten target questions (Table 1) were divided into three categories based on their topic (PET – suitability of chimpanzees as pets, ENTERTAINMENT – presence of chimps in the media, ENDANGERED – survival of chimpanzees in the wild) and a composite score was calculated for each question category. Different question formats were collapsed in each category by using binomial coding of correct answers in both multiple choice and yes/no questions while response scales were transformed from a 1 – 5 scale to a 0 – 1 scale. The composite scores and individual scale questions were analyzed using one-way ANOVAs and post-hoc Tukey tests to evaluate differences between conditions. The exception to this was individual questions with binomial responses which were analyzed using Chi Square tests. All statistical analyses were computed in SPSS, version 18.

### Experiment 2

#### Data Collection

Experiment 2 was designed to replicate and extend the findings of Experiment 1 with a larger sample. Duke University students and affiliated members were recruited through the Fuqua School of Business's online experiment sign-up registry. Seven sessions of 7 – 16 participants were conducted between March and April 2010 (n = 133). Sessions proceeded identically to those in Experiment 1 but ended with a funneled debriefing following the donation period to assess participants' knowledge of the study aim.

#### Videos

The videos used in Experiment 2 were identical to those in Experiment 1.

#### Survey

In Experiment 2 the survey was similarly structured but refined to 1) delete questions that proved superfluous in Experiment 1 2) strengthen the link between questions and the video stimuli and 3) reworded to increase response rates. The revised survey contained 70 questions: 11 target questions on attitudes and knowledge towards chimpanzees, 33 questions about the decoy videos and 26 control questions assessing subjects' demographics, access to information, donation history and environmental habits. To assess participants' hypothetical desire to donate to conservation we added one question asking participants to split $50 between the American Red Cross and the World Wildlife Fund. As an additional control, following the donation period, participants were given a funneled debriefing questionnaire where they were asked to state the aim of the study in five questions [27]. Only three participants (2.2%) correctly identified the aim of the study and were excluded from all analyses.

#### Donations

To increase the likelihood of donations in Experiment 2, we removed the option to donate to the Red Cross and chose a conservation organization with a broader aim: the African Wildlife Foundation. In addition, only 12 oz cans of Coca-Cola were available for purchase. Other aspects of the donation/purchase methodology remained identical to Experiment 1.

#### Analysis

Analyses were performed identically to those in Experiment 1.

## Results

### Experiment 1

**Figure 1:**
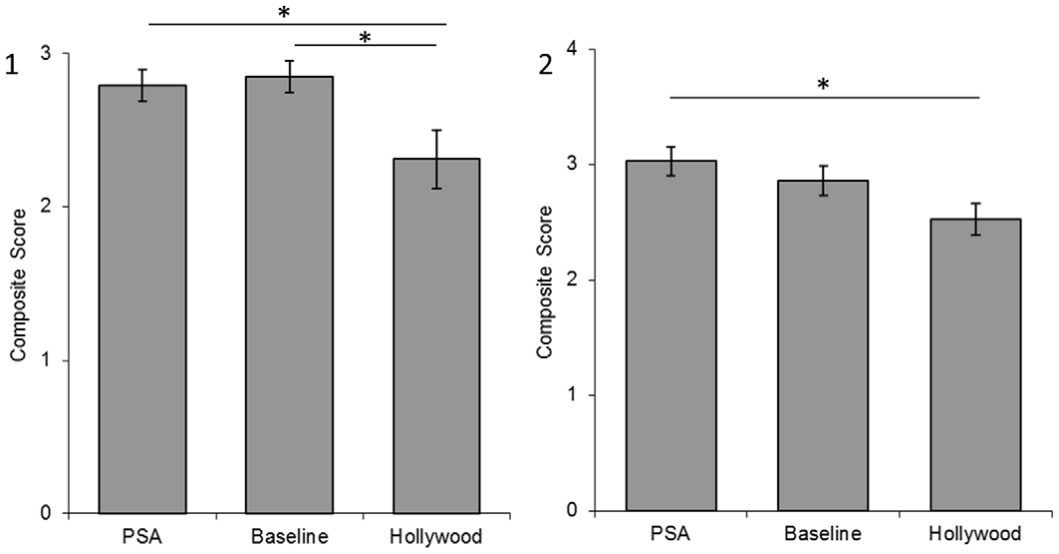
Pet composite scores from Experiment 1 (1) and Experiment 2 (2). A score of 3 in Experiment 1 & a score of 4 in Experiment 2 indicates total agreement that chimpanzees do not make good pets. Error bars represent the standard error of the mean. Significant differences between groups are represented with * p<.05.

**Figure 2:**
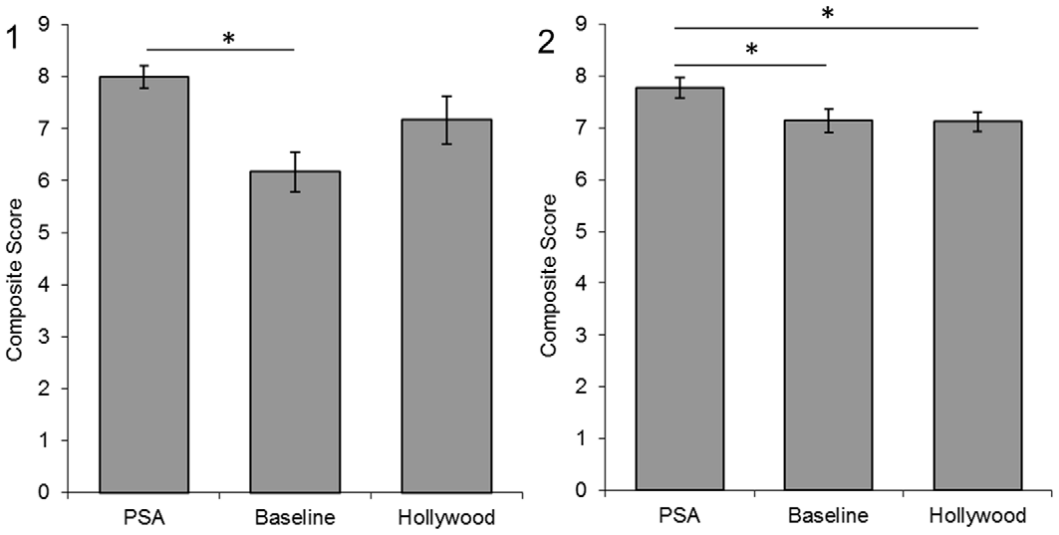
Entertainment composite scores from Experiment 1 (1) and Experiment 2 (2). A score of 9 indicates understanding of the situation facing chimpanzees in entertainment. Error bars represent the standard error of the mean. Significant differences between groups are represented with * p<.05.

**Figure 3:**
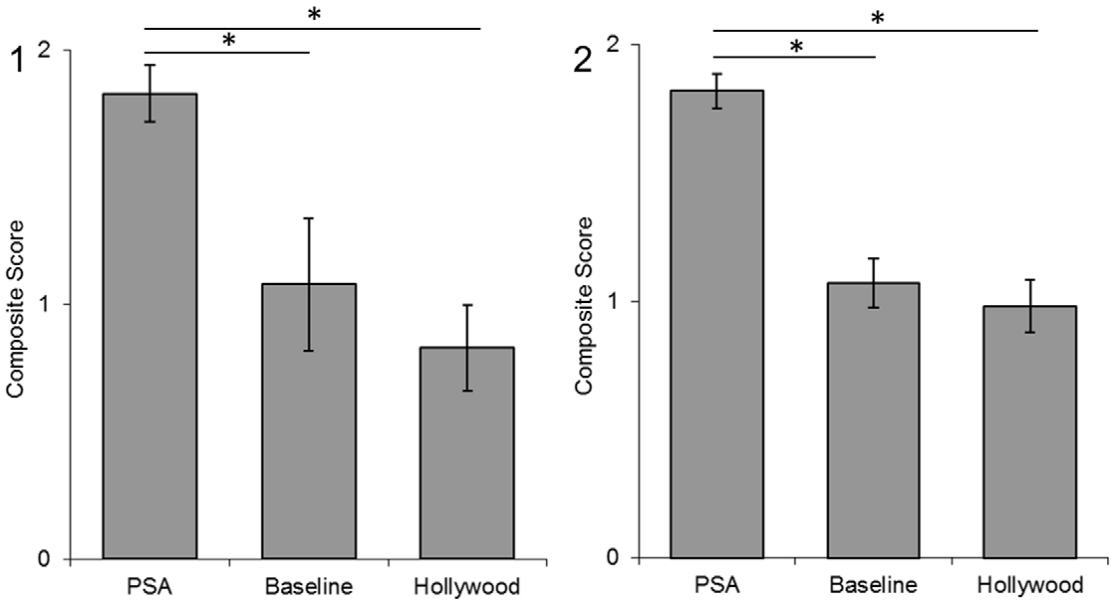
Endangered composite scores from Experiment 1 (1) and Experiment 2 (2). A score of 2 indicates a correct understanding of the population size of wild chimpanzees. Error bars represent the standard error of the mean. Significant differences between groups are represented with * p<.05.

The PET composite score (Figure 1) differed between conditions (F  =  4.68, p  =  .016) and Tukey post-hoc tests indicate (p<.05) participants in the PSA and Baseline conditions showed a better understanding of the suitability of chimpanzees as pets than those in the Hollywood condition. The ENTERTAINMENT composite score (Figure 2) differed between conditions (F  =  6.26, p  =  .005) and post-hoc tests indicate (Tukey tests, p<.01) participants in the PSA condition had a higher strength of agreement that chimpanzees are not suitable for use in the media than the Baseline condition while Hollywood did not. The ENDANGERED composite score (Figure 3) also differed between conditions (F  =  7.53, p  =  .002) and post-hoc tests indicate (Tukey tests, p<.05) participants in the PSA condition responded more accurately about the wild status of chimpanzees than the Baseline and Hollywood conditions.

**Figure 4:**
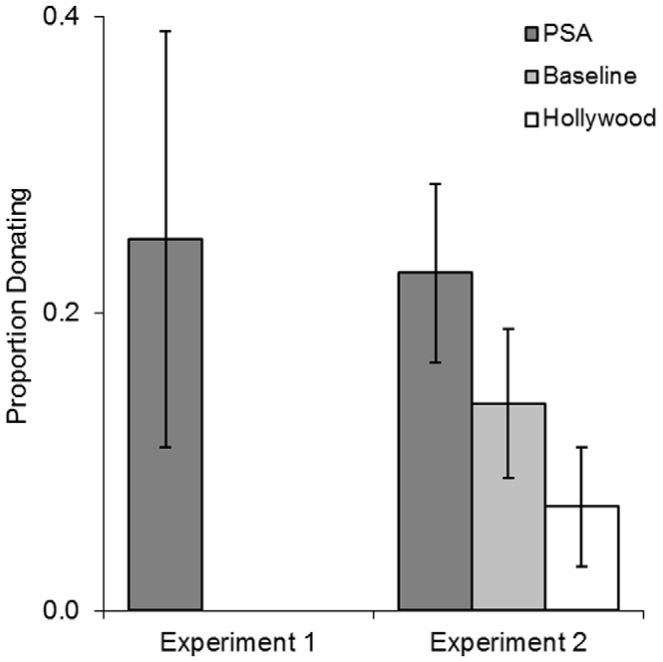
Proportion of subjects donating to an African conservation organization. Proportion of all participants donating to the conservation organization (Experiment 1: Bushmeat Crisis Task Force, Experiment 2: African Wildlife Foundation) in each condition. Error bars represent the standard error of the mean.

When given the opportunity to donate money to Bushmeat Crisis Task Force (BCTF) and/or the American Red Cross, 3 participants donated to the BCTF and 7 participants donated to the American Red Cross. In the PSA condition, three participants donated a combined $8 to the BCTF and 4 participants donated a combined $11 to the Red Cross. Importantly, no participants donated to BCTF in the Baseline or Hollywood conditions (Figure 4).

Though participants were assigned to conditions randomly there was a bias towards individuals with a completed bachelor's degree in the Hollywood condition. Otherwise, all other control questions did not differ across conditions (Table S1).

### Experiment 2

PET composite scores (Figure 1) differed by condition (F  =  3.82, p  =  .04) and post-hoc tests indicate (p<.05) participants in the PSA condition showed a better understanding of the suitability of chimpanzees as pets in comparison to the Hollywood condition. The ENTERTAINMENT composite score (Figure 2) differed by condition (F  =  3.30, p  =  .04) and post-hoc tests indicate a strong trend (Tukey test, <.07) for participants in the PSA condition to have a higher strength of agreement that chimpanzees are unsuitable for use in the media than the Baseline and Hollywood conditions. The ENDANGERED composite score (Figure 3) also differed by condition (F  =  26.62, p<.001) and post-hoc tests (Tukey test, p<.001) indicate participants in the PSA condition responded more accurately about the status of the wild population than the Baseline and Hollywood conditions.

When asked to allocate a hypothetical $50 between the World Wildlife Fund and the Red Cross, participants in the PSA condition allocated the most money to the WWF, followed by the Baseline and Hollywood conditions (see Table 1 for mean donation rate). However, these preferences did not differ significantly by condition (F  =  1.27, p  =  .284). When given the opportunity to donate actual money they had earned in the experiment to the African Wildlife Foundation, 19 participants donated. Both the proportion of participants donating and the amount donated was more than twice as high in the PSA condition when compared to the Hollywood condition (Figure 4). Ten participants donated a combined $23 in the PSA condition, 6 participants donated a combined $22 in the Baseline condition and 3 participants donated a combined $9 in the Hollywood condition.

Though participants were assigned to conditions randomly, the sex ratio in our sample was not equal. The Hollywood condition contained more females than the other conditions (F  =  8.27, p  =  .016). All other control questions did not differ across conditions (Table S1).

To obtain a measure of the likeability for commercials featuring chimpanzees, respondents rated their preference for commercials featuring music, historical places, pets, athletes, famous actors, sports events, animated animals, wild animals and chimpanzees in human-like situations. There was no difference in preference across commercial types in the three conditions so responses were combined for further analysis. Participants preferences differed between commercial type (F  =  13.6, p<.001) and post hoc tests indicate (Tukey test, p<.03) participants favored commercials featuring music to all others, and disliked commercials featuring chimpanzees in human like situations compared to all others except sporting events (Figure S1).

## Discussion

The results of our two experiments provide strong support for the distortion hypothesis and no support for the familiarity hypothesis. Participants watching the conservation message were more likely to indicate that chimpanzees are endangered and unsuitable as pets or entertainers than those viewing the natural behavior of chimpanzees or commercials utilizing trained chimpanzees. When those watching the entertainment chimpanzees differed from the baseline condition, they only showed a significantly reduced understanding of chimpanzee conservation and welfare concerns.

Contrary to the familiarity hypothesis, there was no positive effect of chimpanzee commercials. Perhaps most alarming is the finding that over 35% of those watching entertainment condition thought private citizens should have the right to own a chimpanzee as a pet - in comparison to 10% in the other conditions. This increase in approval is likely related to misperceptions created by chimpanzee commercials about the size, desirability and abundance of chimpanzees. Advertisers only use easily manageable young chimpanzees in commercials but based on our survey viewers believe these chimpanzees were adults – leaving them unaware of how dangerous these animals can be when fully grown. Such a frivolous use of chimpanzees also leads those watching chimpanzee commercials to overestimate their population size in the wild. Clearly, chimpanzee commercials violated participants' expectations about how perilously endangered animals are treated. This confusion likely explains why those watching commercials including entertainment chimpanzees donated the least of their experimental earnings to a conservation charity. Perhaps most damaging to those using chimpanzees in commercials is the stronger preference by participants for all forms of marketing other than those using chimpanzees (e.g. commercials including music, sports stars, etc.). Given the strong support the distortion hypothesis received here, the greater preference participants showed for other marketing techniques, and previously stated welfare concerns for individual chimpanzee actors it is clear that chimpanzees should no longer be used as a marketing tool or for entertainment purposes.

Both studies were designed with a number of controls to rule out possible confounds in the make-up of our subject pool, the differing background knowledge of participants, and the detection of the purpose of the study. First, because gender, altruistic tendencies, educational background and residence patterns (urban/rural) could influence our results if variance in such traits were not equally distributed across our three test groups, we confirmed that all such variables were balanced across the groups in both experiments. The exceptions to this balance could only have worked against the distortion hypothesis (i.e. more women and college graduates in the Hollywood condition) [28], [29]. Second, to avoid participants detecting the purpose of the study we framed the research in broad terms as a marketing study. Our target videos were embedded in a series of decoy commercials and our between subjects design prevented any one subject from seeing all of these test videos. Our survey also included far more questions regarding the different decoy videos than the actual target videos. These methods of camouflage appear to have worked well, as only 2% of subjects correctly identified chimpanzees as the focus of the research when explicitly asked in experiment 2. Therefore, our results are not due to experimental demand or Hawthorne effects, but represent valid measures of knowledge and attitudes.

Our results underscore the potential power of research into human conservation psychology [30]. As the tepid support for climate legislation in the U.S. in the face of overwhelming evidence for the need for action demonstrates, it is not enough for scientists to simply describe how nature operates if facts are to inform conservation decisions [31]. Careful study of how conservation messages must be crafted will always be needed. The conservation message used in the current study is an example of how more careful messaging could avoid unintended effects and increase the impact of the message. Our results show that participants who watched the conservation message incorrectly assumed the private ownership of chimpanzees is illegal in the United States. This means individuals who might care most about ending the ape pet trade are unlikely to act since they incorrectly assume endangered animals cannot be pets. Because this issue is confusing, future messages must make clear that there are no federal laws against primate pet ownership in the United States (only 23 states fully ban private ownership of chimpanzees). In addition, while almost all subjects understood chimpanzees are endangered after watching the conservation message (up from a dismal 50% in the baseline), this understanding did not generalize to other species of great apes. Therefore, to have the greatest impact, public service announcements about a flagship species must also mention the status of other closely related species. Finally, the current research highlights the importance of assessing donations preferences with behavioral measures in addition to simple hypothetical questions in future research. After viewing the conservation message, in experiment 1 subjects indicated they would donate to human centered charities before an animal charity. However, when given the opportunity to donate actual money just earned in the experiment, subjects who watch the conservation commercial donated almost as much to the conservation organization as to the Red Cross. Survey questions regarding donations preferences cannot be viewed as reliable until first validated by actual donation behavior.

While the current results point to future avenues of research they also clearly show that the presentation of chimpanzees in the popular media can have a major impact in distorting the publics' view of chimpanzees. Unfortunately this distortion may have a devastating effect on great ape conservation efforts. These commercials likely confuse the U.S. general public, as they did our relatively well educated sample of subjects – leading to decreased attention or support for ape conservation. Worse yet, media campaigns originating in the U.S. have enormous influence over viewers in less developed countries. The depiction of westerners on television or in print media interacting with chimpanzees as if they are pets can only further encourage the belief of poachers and animal traffickers that expatriates and foreigners have a strong desire for infant chimpanzees [15]. Crashing ape populations that reproduce so slowly will not recover from even a small increase in demand – whether the result of a real or perceived demand for these animals. Therefore, conservation of the great apes in their range countries may begin outside their range countries by finding ways to end the use of privately owned chimpanzees as pets and entertainers.

## Supporting Information

Figure S1 **Mean responses from all conditions combined to the question ‘I enjoy commercials that feature…”.** Scored on a 1 – 5 scale with 1 indicating strong disagreement and 5 indicating strong agreement. Post hoc Tukey tests indicate music is preferred to all other types, and human-like chimps are disliked in comparison to all other types except sports events. Error bars represent the standard error of the mean.
(TIF)

Table S1 **Control Question Results from Experiments 1 and 2.** Summary of answers to control questions. Question format and statistical results are indicated for Experiment 1 & Experiment 2. Format: Y/N – Yes or No question, Scale 1 – 5 – Rate answer on a fixed scale, MC – multiple choice, DC – dichotomous choice, NR – numerical response. – indicates question was not asked in experiment. Result: P – PSA condition, B – Baseline condition, H – Hollywood condition, NS – not significant.
(DOC)
