# Supplementary figures and images for: Use of “Entertainment” Chimpanzees in Commercials Distorts Public Perception Regarding Their Conservation Status
Source: PLoS One. 2011 Oct 12;6(10):e26048. doi: 10.1371/journal.pone.0026048 (PMC3192158; doi:10.1371/journal.pone.0026048)

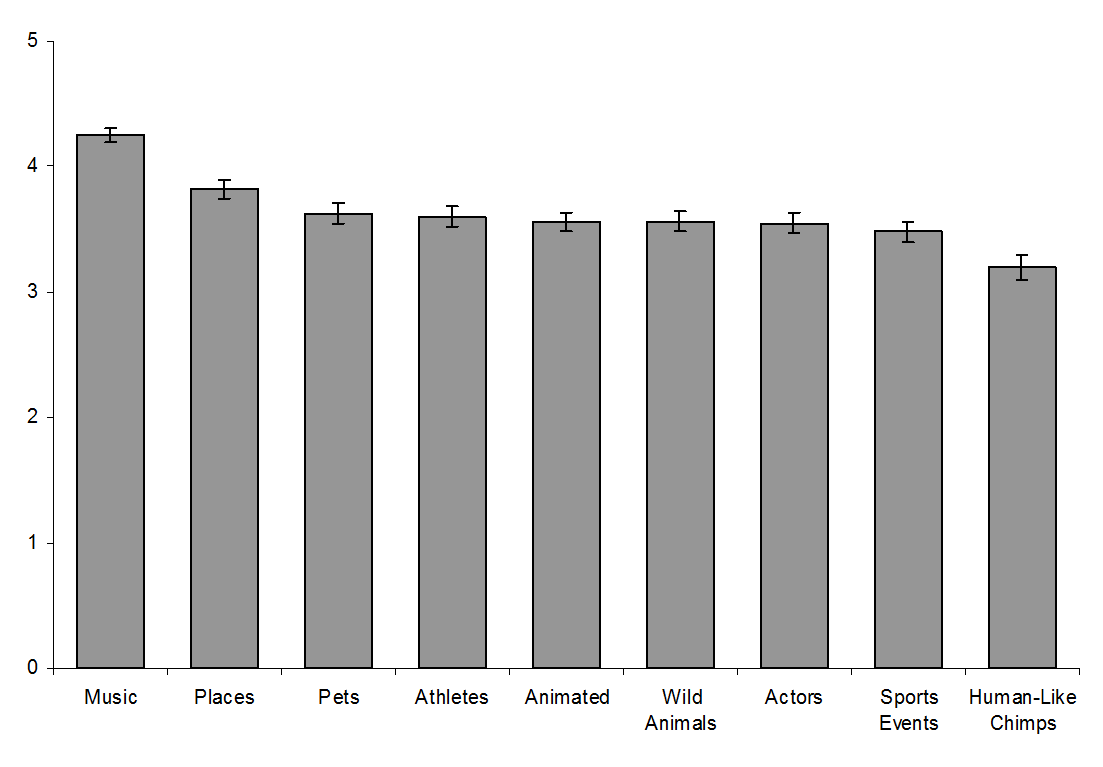

Supplement: Figure S1 — Mean responses from all conditions combined to the question ‘I enjoy commercials that feature…”. Scored on a 1 – 5 scale with 1 indicating strong disagreement and 5 indicating strong agreement. Post hoc Tukey tests indicate music is preferred to all other types, and human-like chimps are disliked in comparison to all other types except sports events. Error bars represent the standard error of the mean. (TIF) [file pone.0026048.s001.tif]
