# Supplementary material for: Use of “Entertainment” Chimpanzees in Commercials Distorts Public Perception Regarding Their Conservation Status
Source: PLoS One. 2011 Oct 12;6(10):e26048. doi: 10.1371/journal.pone.0026048 (PMC3192158; doi:10.1371/journal.pone.0026048)
Supplement: Table S1 — Control Question Results from Experiments 1 and 2. Summary of answers to control questions. Question format and statistical results are indicated for Experiment 1 & Experiment 2. Format: Y/N – Yes or No question, Scale 1 – 5 – Rate answer on a fixed scale, MC – multiple choice, DC – dichotomous choice, NR – numerical response. – indicates question was not asked in experiment. Result: P – PSA condition, B – Baseline condition, H – Hollywood condition, NS – not significant. (DOC) [file pone.0026048.s002.doc]

**Table S**1: Control Question Results from Experiments 1 & 2

| Question | Format | Exp 1 | Exp 2 |
| --- | --- | --- | --- |
| **DEMOGRAPHICS** |  |  |  |
| Sex | M/F | NS | H<B, H < P |
| Age | NR | NS | NS |
| Education | MC | H<B, H<P | NS |
|  |  |  |  |
| **ACCESS TO INFORMATION** |  |  |  |
| How often do you read the newspaper? | Scale 1 – 5 | NS | NS |
| How many hours of TV do you watch per week? | NR | NS | NS |
| How often do you watch nature documentaries? | Scale 1 – 5 | NS | NS |
| How often do you visit web sites run by charity organizations? | Scale 1 – 5 | NS | NS |
|  |  |  |  |
| **LIFESTYLE** |  |  |  |
| Have you ever traveled to a LDC? | Y/N | NS | NS |
| Travel in primate regions? | - | NS | NS |
| How many times have you traveled to a National Park in the US? | NR | NS | - |
| Have you ever traveled to a National Park abroad? | Y/N | NS | - |
| Do you or your family have any exotic pets? | Y/N | NS | NS |
| How often have you visited a zoo or animal sanctuary in the last year? In the last 5 years? | MC | NS | NS |
| How often do you volunteer for environmental organizations? | Scale 1 – 5 | NS | NS |
|  |  |  |  |
| **KNOWLEDGE** |  |  |  |
| Which of the following are Great Apes: Chimpanzee | Y/N | NS | NS |
| Which of the following are Great Apes: Bonobos | Y/N | NS | NS |
| Which of the following are Great Apes: Gorillas | Y/N | NS | NS |
| Which of the following are Great Apes: Orangutans | Y/N | NS | NS |
|  |  |  |  |
| **DONATION HISTORY** |  |  |  |
| Personal Altruism Scale (Tankersley et al. 2006; 16 questions) | Scale 1 – 5 | NS | NS |
| Red Cross Donation Amount | NR | NS | - |
| Red Cross Donation | Y/N | NS | - |
| Do you agree: I am more likely to donate to a charity if I see a commercial first. | Scale 1 – 5 | NS | NS |
| Which charity are you most/least likely to donate to? | MC* | NS | NS |
| List of past charities donated to | Checkbox | NS | NS |

Summary of answers to control questions. Question format, and statistical results are indicated for Experiment 1 & Experiment 2.

Format: Y/N – Yes or No question, Scale 1 – 5 – Rate answer on a fixed scale, MC – multiple choice, DC – dichotomous choice, NR – numerical response. – indicates question was not asked in experiment. Result: P – PSA condition, B – Baseline condition, H – Hollywood condition , NS – not significant
